# White matter DNA methylation profiling reveals deregulation of *HIP1*, *LMAN2*, *MOBP*, and other loci in multiple system atrophy

**DOI:** 10.1007/s00401-019-02074-0

**Published:** 2019-09-18

**Authors:** Conceição Bettencourt, Sandrine C. Foti, Yasuo Miki, Juan Botia, Aparajita Chatterjee, Thomas T. Warner, Tamas Revesz, Tammaryn Lashley, Robert Balazs, Emmanuelle Viré, Janice L. Holton

**Affiliations:** 1 The Queen Square Brain Bank for Neurological Disorders, UCL Queen Square Institute of Neurology, London, UK; 2 Department of Clinical and Movement Neurosciences, UCL Queen Square Institute of Neurology, London, UK; 3 Department of Neurodegenerative Disease, UCL Queen Square Institute of Neurology, London, UK; 4 Department of Neuropathology, Institute of Brain Science, Hirosaki University Graduate School of Medicine, Hirosaki, Japan; 5 Departamento de Ingeniería de la Información y las Comunicaciones, Universidad de Murcia, Murcia, Spain; 6 Reta Lila Weston Institute of Neurological Studies, UCL Queen Square Institute of Neurology, London, UK; 7 Institute of Prion Diseases, MRC Prion Unit At UCL, Courtauld Building, 33 Cleveland Street, London, UK

**Keywords:** MSA, Pathological subtypes, Neurodegeneration, Brain tissue, EWAS, WGCNA

## Abstract

Multiple system atrophy (MSA) is a fatal late-onset neurodegenerative disease. Although presenting with distinct pathological hallmarks, which in MSA consist of glial cytoplasmic inclusions (GCIs) containing fibrillar α-synuclein in oligodendrocytes, both MSA and Parkinson’s disease are α-synucleinopathies. Pathologically, MSA can be categorized into striatonigral degeneration (SND), olivopontocerebellar atrophy (OPCA) or mixed subtypes. Despite extensive research, the regional vulnerability of the brain to MSA pathology remains poorly understood. Genetic, epigenetic and environmental factors have been proposed to explain which brain regions are affected by MSA, and to what extent. Here, we explored for the first time epigenetic changes in post-mortem brain tissue from MSA cases. We conducted a case–control study, and profiled DNA methylation in white mater from three brain regions characterized by severe-to-mild GCIs burden in the MSA mixed subtype (cerebellum, frontal lobe and occipital lobe). Our genome-wide approach using Illumina MethylationEPIC arrays and a powerful cross-region analysis identified 157 CpG sites and 79 genomic regions where DNA methylation was significantly altered in the MSA mixed-subtype cases. *HIP1*, *LMAN2* and *MOBP* were amongst the most differentially methylated loci. We replicated these findings in an independent cohort and further demonstrated that DNA methylation profiles were perturbed in MSA mixed subtype, and also to variable degrees in the other pathological subtypes (OPCA and SND). Finally, our co-methylation network analysis revealed several molecular signatures (modules) significantly associated with MSA (disease status and pathological subtypes), and with neurodegeneration in the cerebellum. Importantly, the co-methylation module having the strongest association with MSA included a CpG in *SNCA*, the gene encoding α-synuclein*.* Altogether, our results provide the first evidence for DNA methylation changes contributing to the molecular processes altered in MSA, some of which are shared with other neurodegenerative diseases, and highlight potential novel routes for diagnosis and therapeutic interventions.

**Electronic supplementary material:**

The online version of this article (10.1007/s00401-019-02074-0) contains supplementary material, which is available to authorized users.

## Introduction

Multiple system atrophy (MSA) is an incurable progressive neurodegenerative disease of adult-onset. Clinically, patients present with an atypical parkinsonian syndrome, with little or no response to levodopa, cerebellar ataxia and autonomic dysfunction. Neuropathological examination reveals regional neuronal loss with specific glial and neuronal inclusions containing fibrillar α-synuclein. Like Parkinson’s disease (PD), MSA is an α-synucleinopathy. Although these two diseases may overlap clinically they differ pathologically, with PD α-synuclein aggregates forming Lewy bodies in neurons, in MSA the pathological hallmark is the presence of oligodendroglial glial cytoplasmic inclusions (GCIs) containing aggregated α-synuclein, with additional α-synuclein accumulation forming less frequent neuronal cytoplasmic inclusions (NCIs) [26, 35, 46]. GCIs are important in the pathogenesis of MSA as their number correlates with disease duration and also with the severity of neurodegeneration [33]. The regional distribution of pathological changes underlies the clinical symptoms of MSA. Patients with a prominent parkinsonian movement disorder have predominantly striatonigral degeneration (SND subtype) and those in which cerebellar signs predominate have more severe olivopontocerebellar atrophy (OPCA subtype) [33, 54]. At post-mortem examination cases often show an equal distribution of neurodegeneration in SND and OPCA regions giving rise to the mixed subtype (SND = OPCA) [33]. Other brain regions, including the sub-cortical white matter show variable vulnerability to GCI pathology. The mechanisms determining oligodendrocyte vulnerability to α-synuclein aggregation and the susceptibility of different brain regions to pathological changes are unknown. However, the existence of geographical variation in the predominance of the MSA pathological subtypes, with OPCA predominating in the Japanese population and SND being more common in Caucasians, strongly suggests that genetic, epigenetic and/or environmental factors are playing a role in the regional vulnerability to the disease process [34].

In recent years, epigenetic modifications such as DNA methylation changes have been identified in neurodegenerative diseases, including Alzheimer’s disease (AD) and PD (e.g. [5, 6, 28, 42, 44, 53]). Epigenetic modifications represent molecular regulatory mechanisms through which environmental and lifestyle factors, in combination with the individual genetic makeup, may modulate the risk of disease, leading to an increasing interest in investigating their role in human diseases. We hypothesized that the selective vulnerability of different brain regions to α-synuclein pathology and neurodegeneration in MSA may involve differential DNA methylation. To investigate this, we have analysed genome-wide DNA methylation profiles in MSA and controls using the Infinium MethylationEPIC BeadChip (Illumina), which examines over 850,000 methylation sites per sample at single-nucleotide resolution across the whole-genome. First, we have selected regions with varying severity of GCI pathology in MSA: a severely affected region (cerebellar white matter), a moderately affected region (frontal white matter) and a region with little involvement (occipital white matter), and performed an epigenome-wide association study (EWAS, MSA mixed subtype versus controls—discovery cohort). Next, we have expanded the number of MSA cases to include the three pathological subtypes (OPCA predominant, SND predominant and mixed) and controls, and investigated in cerebellar white matter the DNA methylation changes identified in the discovery cohort of MSA mixed-subtype cases. To our knowledge this is the first study investigating genome-wide epigenetic changes in brain regions affected in MSA.

## Materials and methods

### Demographic and clinical characteristics of post-mortem brain donors

All tissue came from brains donated to the Queen Square Brain Bank archives, where it is stored under a licence from the Human Tissue authority (No. 12198). The brain donation programme and protocols have received ethical approval for donation and research by the NRES Committee London-Central. All cases were characterized by age, gender, disease history (including disease onset and duration) as well as neuropathological findings. Apart from frozen tissue, formalin-fixed paraffin-embedded (FFPE) sections were also available for detailed neuropathological evaluations, including sections stained for standard haematoxylin and eosin (H&E) and with immunohistochemistry for α-synuclein [24]. FFPE sections from 9 MSA mixed subtype and 6 normal control donors matching the discovery cohort described below (Supplementary Table S1, Online Resource 1) were also used for the analysis of global DNA methylation.

**Fig. 1:**
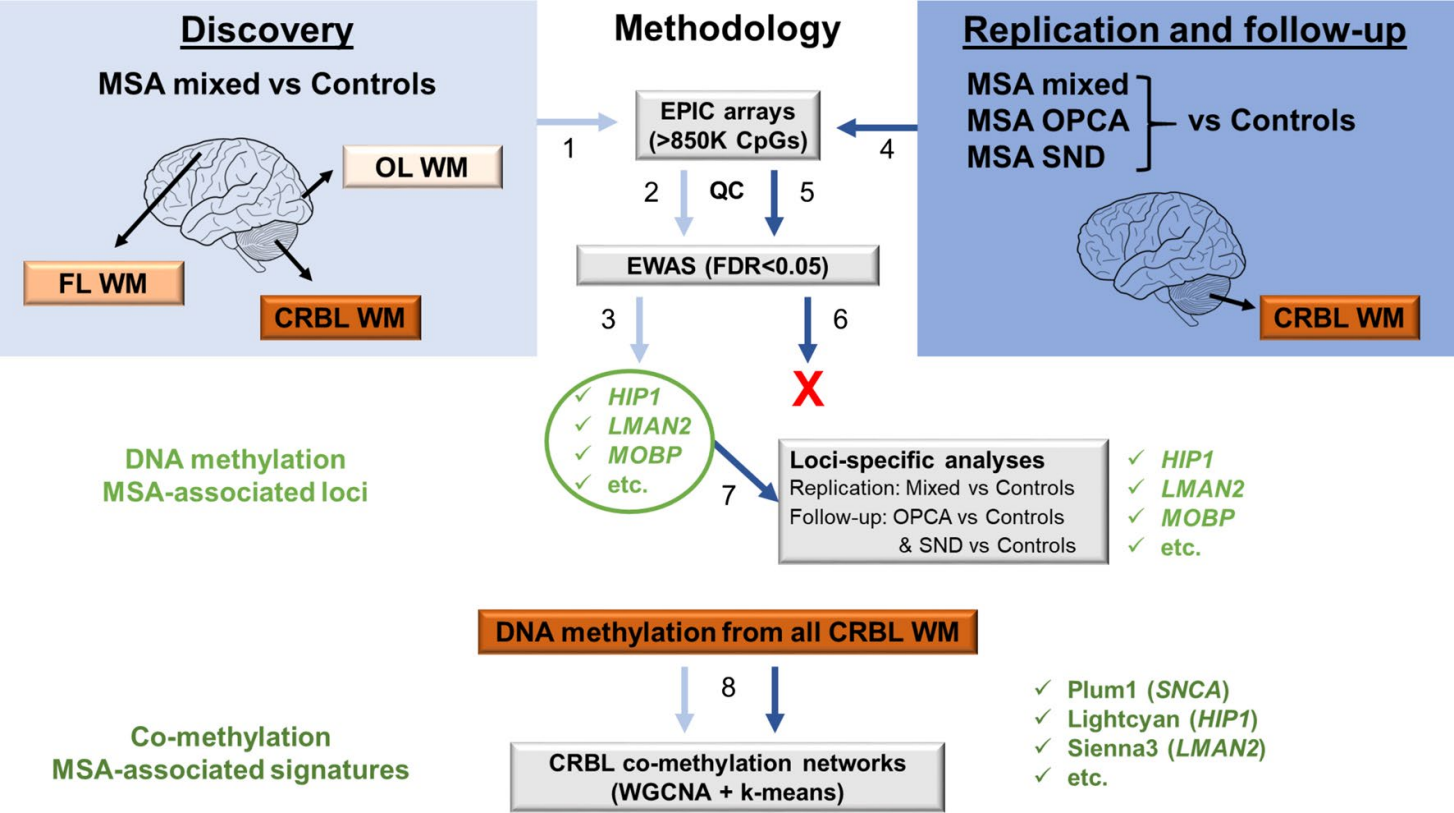
Flowchart of the analysis process in a multi-stage multiple system atrophy (MSA) study. Briefly, for the discovery cohort, DNA extracted from white matter tissue of three brain regions (*N* = 48 samples in total) was used for DNA methylation profiling using the Illumina MethylationEPIC arrays (covering > 850,000 methylation sites) (**1**). The EPIC array data were subject of thorough quality control (**2**, 47 samples and 756,224 CpG sites were used for downstream analyses). To identify DNA methylation MSA-associated loci, we have then performed an epigenome-wide association study (EWAS) comparing MSA mixed-subtype cases to controls, and accounting for possible confounder factors (e.g. age, sex, post-mortem delay, and neuronal proportions). The EWAS was done for individual CpGs (significance threshold FDR < 0.05) as well as for larger genomic regions comprising several CpGs (significance threshold Stouffer < 0.05). Some of the genes, which showed significant differences in beta values are indicated (**3**). We sought to replicate the results from the discovery cohort in the replication and follow-up cohort that comprised a second MSA mixed-subtype cohort as well as a set of the other MSA pathological subtypes (OPCA and SND subtypes). DNA extracted from cerebellar white matter tissue only (*N* = 48 samples in total) was used for DNA methylation profiling using the Illumina MethylationEPIC arrays (**4**), which was subject of thorough quality control checks (**5**, 46 samples and 758,752 CpG sites were used for downstream analyses). The EWAS comparing each MSA subtype to controls, and accounting for possible confounder factors (e.g. age, sex, post-mortem delay, and neuronal proportions), did not reveal significant DNA methylation changes at the genome-wide level (**6**). As our aim was to replicate the MSA mixed-subtype-associated loci identified in the discovery cohort in a new cohort of MSA mixed-subtype cases and to follow-up these loci in the other two MSA pathological subtypes (OPCA and SND), we then performed loci-specific analyses in the second cohort (**7**). Finally, we have used an alternative analysis approach by applying weighted gene correlation network analysis to the 10% most variable CpG sites (*N* = 75,798 CpG sites) from all cerebellar samples (*N* = 62, from both cohorts), to identify co-methylation MSA-associated signatures (**8**). *MSA* multiple system atrophy, *OPCA* olivopontocerebellar atrophy, *SND* striatonigral degeneration, *CRBL* cerebellum, *FL* frontal lobe, *OL* occipital lobe, *WM* white matter, *QC* quality control, *EWAS* epigenome-wide association study, *FDR* false discovery rate, *WGCNA* weighted gene correlation network analysis

For our epigenome-wide scan, we have investigated DNA methylation changes in samples from a total of 64 post-mortem brain donors [42 MSA donors (mean age 64.3 ± 7.65 years, gender = 21 males/21 females); and 22 neurologically normal control donors (mean age 80 ± 8.93 years, gender = 11 males/11 females)]. Data were generated in a multi-phase study design (Fig. 1). To minimize any bias due to neuropathological differences across MSA subtypes, only mixed (SND = OPCA) cases were selected for the discovery phase. Also, given the selective vulnerability of certain brain regions in MSA, at this phase of the study, we selected three brain regions per donor with varying severity of GCI pathology: a severely affected region (cerebellar white matter), a moderately affected region (frontal white matter) and a region with little involvement (occipital white matter), while for the follow-up phase we have investigated the cerebellar white matter only (Supplementary Table S1, Online Resource 1). We have dissected white matter from each brain region to enrich for oligodendrocytes in which most of the aggregated α-synuclein is found in MSA. Our discovery cohort included 10 pathologically confirmed MSA donors classified as MSA mixed subtype (mean motor onset 57.1 ± 6.57 years; mean disease duration 7 ± 3.80 years) and 6 neurologically normal controls. Our follow-up cohort is composed of 16 additional MSA mixed-subtype cases (mean motor onset 55.5 ± 7.54 years; mean disease duration 9.67 ± 12.5 years), 16 controls, 8 MSA OPCA subtype cases (mean motor onset 58.5 ± 11.5 years; mean disease duration 7.25 ± 4.10 years), and 8 MSA SND subtype cases (mean motor onset 57.5 ± 11.6 years; mean disease duration 8.12 ± 2.17 years). Mean age at motor onset and mean disease duration did not differ significantly across MSA cohorts/subtypes. MSA neuropathological subtyping was based on previously described criteria [33].

### Analysis of global methylation by 5-methylcytosine immunohistochemical staining

To investigate global DNA methylation patterns in MSA compared to controls we have used immunohistochemistry as previously described [23]. Briefly, 8-μm-thick FFPE tissue sections were obtained from the cerebellum, frontal lobe and occipital lobe. Heat antigen retrieval pre-treatment was used prior to application of the primary antibody, anti-methylcytosine (anti-5mC 1:250, Abcam Ab10805), followed by incubation with a biotinylated polyclonal secondary anti-rabbit (1:200, DAKO). Sections were then incubated with an avidin–biotin peroxidase complex solution (Vectastatin ABC kit, Vector laboratories) to amplify the signal and then with 3′3′-diaminobenzidine solution (DAB) to visualize the signal. Finally, sections were counterstained with Mayer’s haematoxylin as the nuclear counterstain.

Quantitative analysis of the slides immunostained for 5mC was undertaken to compare global methylation patterns in MSA mixed-subtype versus neurologically normal controls (discovery cohort, except one MSA case for whom no FFPE tissue was available, Supplementary Table S1, Online Resource 1). The brain regions analysed included the cerebellum, posterior frontal lobe and the occipital lobe (white matter only). The areas of interest were marked and positively or negatively stained nuclei assessed by an observer blinded to the diagnosis. The percentage of positively stained nuclei out of the total cell population count was calculated.

Pairwise comparisons between MSA cases and controls for each brain region were performed using the Wilcoxon rank-sum test with Bonferroni correction for multiple testing. Statistical analysis was performed with the R suit and *p* values < 0.05 were considered as significant.

### Infinium EPIC BeadChip methylation profiling and quality control

Genomic DNA was extracted from flash-frozen brain tissue using standard protocols. A bisulfite conversion was performed using 500 ng of genomic DNA using either the TrueMethyl^®^Array Kit (Cambridge Epigenetix, discovery phase) or the EZ DNA Methylation Kit (Zymo Research, follow-up phase). Genome-wide methylation profiling was performed at UCL Genomics, using the Infinium HumanMethylationEPIC BeadChip (Illumina), which covers over 850,000 CpG sites, following the manufacturer’s instructions. Beta values were used to estimate the methylation levels of each CpG site using the ratio of intensities between methylated and unmethylated alleles. Beta values range from 0 to 1, representing approximately 0–100% methylation, respectively.

The analysis of the EPIC array DNA methylation data was performed using several R Bioconductor packages. Briefly, raw data (idat files) were imported and then rigorously pre-processed with minfi [3] and ChAMP packages [49]. Thorough quality control checks were performed with minfi, watermelon [37], and ChAMP packages. Probes that met one or more of the following criteria were excluded from further analysis: (1) poor quality, (2) cross reactive, (3) included common genetic variants, and (4) mapped to X or Y chromosome. Over 750,000 CpG sites were used for downstream analysis. Samples were dropped during quality control if: (1) presenting with high failure rate, (2) the predicted sex was not matching the phenotypic sex, and (3) inappropriately clustering on multidimensional scaling analysis.

### Differential methylation analysis

To assess the magnitude of the differences between comparison groups (MSA versus controls), delta beta values were computed as the difference between the mean beta values in each of the MSA groups (MSA mixed subtype discovery, MSA mixed subtype follow-up, MSA OPCA subtype and MSA SND subtype) and the mean beta values in the corresponding control group (controls discovery or controls follow-up). As recommended by Du et al. [9], we have computed and used *M* values (logistic transformation of the beta values) for all statistical analysis. Figure 1 shows an outline of the analysis process.

## Discovery phase

We have applied multiple linear regression models to identify associations between DNA methylation changes (at specific CpG sites and more extended regions) and MSA. We have accounted for possible confounding factors, including age, sex, post-mortem delay, neuronal proportions (estimated using the CETS package [12]), batch effect (as detected during quality control checks) and surrogate variables (as determined by the SVA package [25]) in our regression models. For the analysis of differentially methylated CpG sites we have used the limma package [41], and for the analysis of differentially methylated regions we have used the DMRcate package [36]. We have performed a powerful cross-region analysis (all brain regions pooled together) [44], and then region-specific analysis comparing methylation levels in each brain region (cerebellum, frontal lobe and occipital lobe) between the MSA mixed-subtype cases and the controls. To account for multiple samples from the same individual (cerebellum, frontal lobe and occipital lobe), we have used the duplicateCorrelation function available in the limma package. False discovery rate (FDR) adjusted *p* values < 0.05 were considered significant. To gain insights into the biology underlying differentially methylated CpGs, gene ontology and pathway enrichment analysis was carried out using kegga and goana functions available in the limma package [41] accounting for the number of probes in the EPIC arrays. For differentially methylated regions, enrichment analysis was performed with default parameter values in gProfiler [40].

## Follow-up phase

Because we observed considerable overlap across brain regions in the discovery phase, only cerebellar white matter samples were analysed in a replication cohort of MSA mixed-subtype cases and controls, and additionally in cases of the other two pathological MSA subtypes (MSA OPCA and MSA SND). We have performed an EWAS with data from all the new cerebellar samples as described above in the discovery phase, adjusting for the following covariates in our linear regression model: age, sex, post-mortem delay, neuronal proportions, and surrogate variables. Taking into account the results from the discovery phase, we have performed both CpG-specific and gene-specific analysis to check which DNA methylation changes were replicated in the new MSA mixed-subtype cohort compared to controls as well as to verify whether these changes also occur in the other MSA subtypes. We have also performed a meta-analysis with data from all MSA mixed-subtype cases and all controls (from both discovery and follow-up), using the same approach described above and adjusting for the following covariates in our linear regression model: age, sex, post-mortem delay, neuronal proportions, batch, and surrogate variables. Nominal as well as analysis-wide corrected *p* values were used as specified in the “Results” section.

### Grading cerebellar pathology

As a proxy for neurodegeneration in the cerebellum, we have evaluated the loss of Purkinje cells. To this end, using H&E stained sections from all MSA cases included in the network analysis (see below), we have performed a semi-quantitative analysis of Purkinje cell depletion based on a 4-point scale (0 = absent; grade 1 = mild; grade 2 = moderate; grade 3 = severe) as previously described [33]. For the same cases, we have also performed α-synuclein immunohistochemical staining (anti-α-synuclein antibody 1:1,500, Thermo Scientific MA1-90342) to assess the severity of GCI pathology in the cerebellar white matter using a semi-quantitative approach as previously described (0 = no inclusions; grade 1 = 1–5 inclusions; grade 2 = 6–19 inclusions; grade 3 = more than 20 inclusions) [33]. Loss of Purkinje cells and the severity of GCI pathology were assessed by a neuropathologist (YM) who was blinded to the pathological diagnoses. These data were used for module–disease trait correlations in the co-methylation network analysis described below.

### Cerebellar co-methylation network analysis

We have used a systems biology analysis approach based on weighted gene correlation network analysis (WGCNA) [22] to identify clusters of highly correlated CpGs (co-methylation modules) in an unsupervised manner (i.e. agnostic of gene ontology). To this end, we have used data from all cerebellar samples (from both discovery and follow-up phases of the study), and focused on the top 10% of CpGs with the highest variance across individuals regardless of the disease status (*N* = 75,798 CpG sites). We have used M values adjusted for age, sex, post-mortem delay, neuronal proportions, batch effect and surrogate variables, and constructed a signed network. Modules were calculated using the blockwiseModules function with a power of 10. Module membership was then reassigned using the applyKMeans function of the CoExpNets package [4].

The CpGs inside each module were represented by a weighted average, the module eigengene (ME), which is formally defined as the first principal component of the module, obtained by a principal component analysis on the CpG methylation values. The MEs were correlated with MSA status, MSA subtypes, and other sample traits, including motor onset, disease duration, Purkinje cell loss, and GCI pathology. We have additionally made use of gene significance (GS) measures to investigate the relevance of each CpG within the network for MSA as described elsewhere [22]. Briefly, GS stands for the correlation between CpG-specific methylation levels and the MSA status, and the higher the absolute value of GSi, the more biological significant is the *i*th CpG for MSA.

For each module, the module membership (MM) was determined as the correlation between each CpG and the ME of the corresponding module. Highly connected CpGs within a module (hub CpGs) present with high MM values to the respective module. In the “Results” section, we refer to hub CpGs as those in the top 10 with the highest MM. To gain insights into the biology underlying MSA-related modules, gene ontology and pathway enrichment analysis for CpGs mapping to genes and with MM > 0.40 was carried out using default parameter values in gProfiler [40].

## Results

### The overall degree of DNA methylation remains unchanged in brain tissue of MSA mixed-subtype cases

**Fig. 2:**
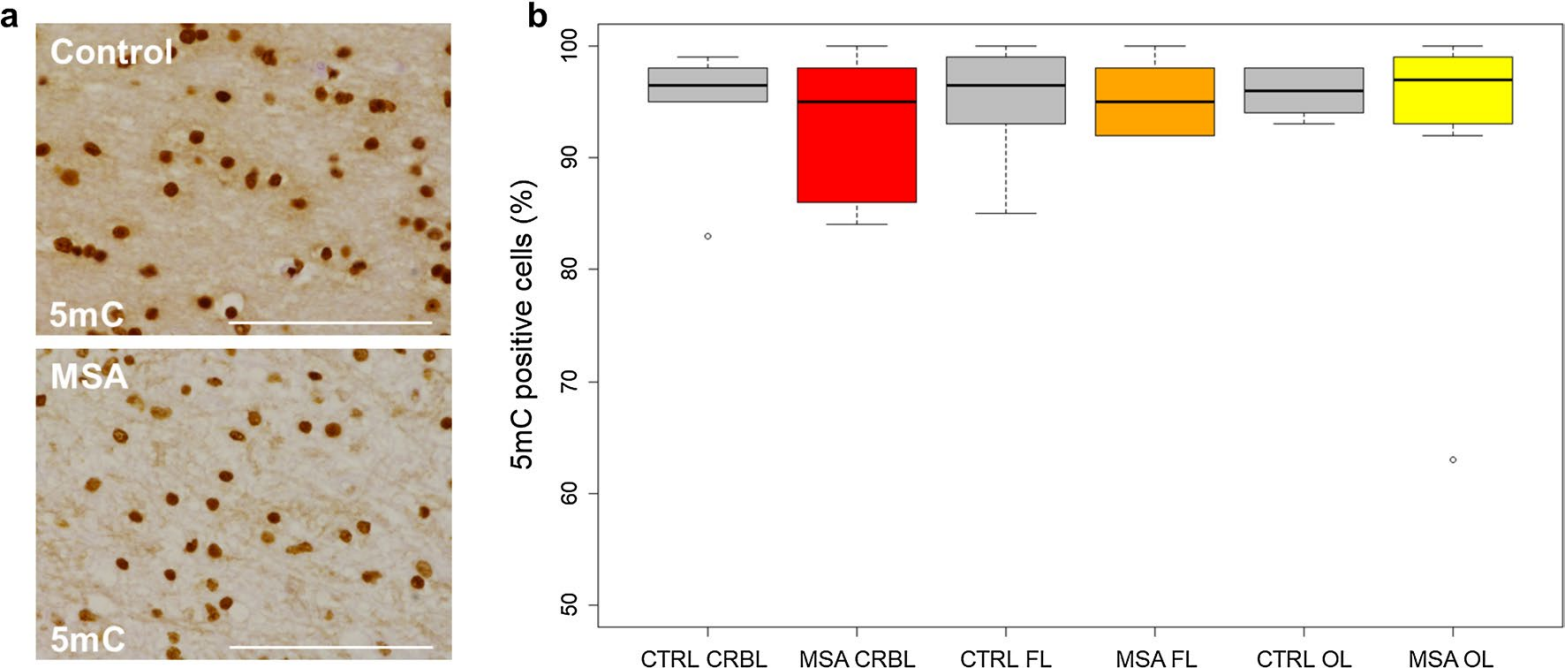
Semi-quantitative analysis of global DNA methylation by immunohistochemistry in multiple system atrophy (MSA) mixed-subtype cases compared to normal controls. **a** 5‐Methylcytosine (5mC) immunohistochemical staining in the white matter of an MSA mixed-subtype case and a normal control (scale bar 100 µm). **b** Boxplot showing no significant differences between MSA mixed-subtype cases (*N* = 9) and controls (*N* = 6) regarding the percentage of cells staining positive for 5mC in three brain regions with distinct degrees of pathology. Grey: control brain samples; red: brain region severely affected in the MSA mixed subtype; orange: brain region moderately affected in MSA; yellow: brain region mildly affected in MSA. *CTRL* control group, *MSA* MSA mixed group, *CRBL* cerebellar white matter, *FL* frontal lobe white matter, *OL* occipital lobe white matter

We first tested whether global DNA methylation patterns are altered in MSA, as it has been reported for other neurodegenerative diseases (e.g. PD and AD [53]). Using an immunohistochemistry approach, we compared the patterns of 5mC staining in MSA mixed-subtype and control cases, in three brain regions with varying severity of GCI pathology in MSA. Figure 2 shows that all three regions display very similar percentages of 5mC positively stained cells in both MSA mixed-subtype cases (*n* = 9) and controls (*n* = 6) (median values in cerebellum and frontal lobe: 96.5% controls vs 95% MSA mixed subtype; median values in occipital lobe: 96% controls vs 97% MSA mixed subtype). These results suggest that potential changes in DNA methylation profiles in MSA cannot be detected globally using immunohistochemistry.

### CpG-specific DNA methylation changes were detected across brain regions in the MSA mixed-subtype discovery cohort

To further explore whether DNA methylation profiles are deregulated in MSA, we next investigated locus-specific DNA methylation changes. To identify specific differentially methylated CpG sites associated with selective pathological vulnerability in MSA, we analysed three brain regions with varying severity of GCI pathology in MSA: cerebellum (severely affected in the MSA mixed subtype), frontal lobe (moderately affected in MSA) and occipital lobe (minimally affected in MSA). From the over 850,000 sites present in the EPIC arrays, 109,884 probes were filtered out during stringent quality control steps. Probes were excluded if presenting poor quality, corresponding to non-CpG sites, overlapping with common SNPs, aligning to multiple locations, and mapping to chromosomes X and Y, leaving a total of 756,224 CpG sites to be used in all downstream analysis.

**Fig. 3:**
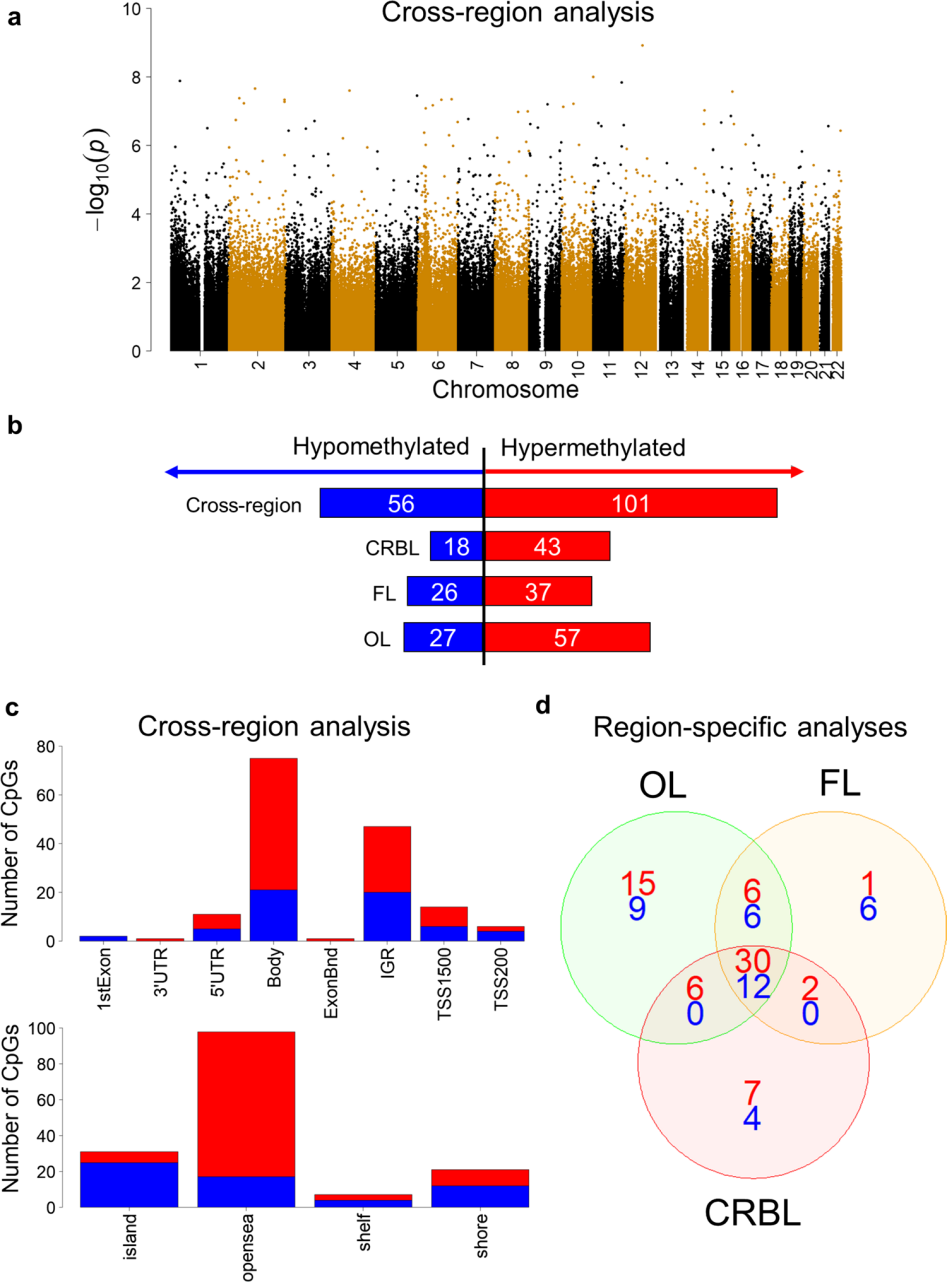
Summary of CpG-specific DNA methylation changes identified during the epigenome-wide association study in multiple system atrophy (MSA) mixed-subtype cases compared to normal controls (discovery phase). **a** Manhattan plot showing the distribution of differentially methylated CpGs across different chromosomes using a powerful cross-region analysis (pooling together samples from cerebellum, frontal lobe and occipital lobe). **b**, **c** Number and distribution of differentially hypo- and hypermethylated CpGs (represented in blue and red bars, respectively) identified in cross-region and region-specific analyses (MSA mixed subtype vs controls, FDR < 0.05). **d** Venn diagram representing the overlap across the three brain regions (cerebellum *CRBL*, frontal lobe *FL*, occipital lobe *OL*), with blue and red numbers indicating the number of CpGs differentially hypo- and hypermethylated in MSA mixed subtype vs controls, respectively

We performed a powerful cross-region analysis by pooling all brain regions together and comparing MSA mixed-subtype cases with controls. This cross-region analysis, identified 157 differentially methylated CpGs mapping across different chromosomes (Fig. 3a), and overlapping with 103 known genes (Supplementary Table S2.1, Online Resource 2). Q-Q plots are shown in Supplementary Fig. 1 (Online Resource 3). These CpGs were mostly hypermethylated, i.e. presented higher levels of methylation in MSA mixed subtype compared to controls (Fig. 3b). Most of the hypermethylated CpGs were located at the gene body or at intergenic regulatory regions (Fig. 3c). CpGs located in CpG islands (commonly at gene promoters) were mostly hypomethylated (Fig. 3c). When comparing the difference in methylation levels at these 157 CpGs between MSA and controls (i.e. delta beta values) for each brain region, the cerebellum (the most affected brain region analysed) presented 80 CpGs with strong changes (absolute delta beta values ≥ 5%). In three of those CpGs (cg04222842-*SRP9*, cg15737168-*TECTA*, cg00720065-intergenic chr 2), such strong effects were observed in the cerebellum but not in the frontal or occipital lobes (absolute delta beta values < 5% in the latter, Supplementary Table S2.1, Online Resource 2), suggesting these are region-specific DNA methylation changes in MSA.

To address region-specific DNA methylation changes further, we have also performed analyses for each brain region separately, comparing MSA mixed-subtype cases against controls for each region. These region-specific analyses revealed 61 differentially methylated CpGs (41 of which with absolute delta beta ≥ 5%) in the cerebellum (mapping to 39 known genes), 63 CpGs in the frontal lobe (44 of which with absolute delta beta ≥ 5%), and 84 CpGs in the occipital lobe (58 of which with absolute delta beta ≥ 5%, Supplementary Tables S2.2–S2.4, Online Resource 2). Those CpGs were mostly hypermethylated in MSA compared to controls (Fig. 3b), and the majority were common across all brain regions (*N* = 42 CpGs) or at least shared by two brain regions (Fig. 3d). Because the cerebellum is the most pathologically affected region amongst the three brain regions we have investigated, we were particularly interested in alterations to DNA methylation profiles in the cerebellum. When comparing MSA mixed-subtype cases and controls, six CpGs (cg15480237 in *SENP6*, cg15463989 in *DGKI,* cg02292205 in *XKR6*, cg22523351 in *NSMAF*, cg04222842 in *SRP9*, and cg09190141 in *HTR3D*; Supplementary Table S2.2, Online Resource 2) showed stronger and significant differences in the cerebellum only (absolute delta beta values ≥ 5%, FDR < 0.05). Importantly, the cg04222842 mapping to the *SRP9* gene showed a gradient of change in DNA methylation levels that mirrors the pathological GCI burden in the three brain regions analysed (− 6.8% in cerebellum, − 4.5% in frontal lobe, and − 2.5% in occipital lobe; Supplementary Table S2.2, Online Resource 2). These results support the existence of brain region-specific changes in MSA, and suggest that the levels of DNA methylation may relate to the GCI pathology burden.

### DNA methylation alterations spanning genomic regions were also detected across brain regions in the MSA mixed-subtype discovery cohort

**Fig. 4:**
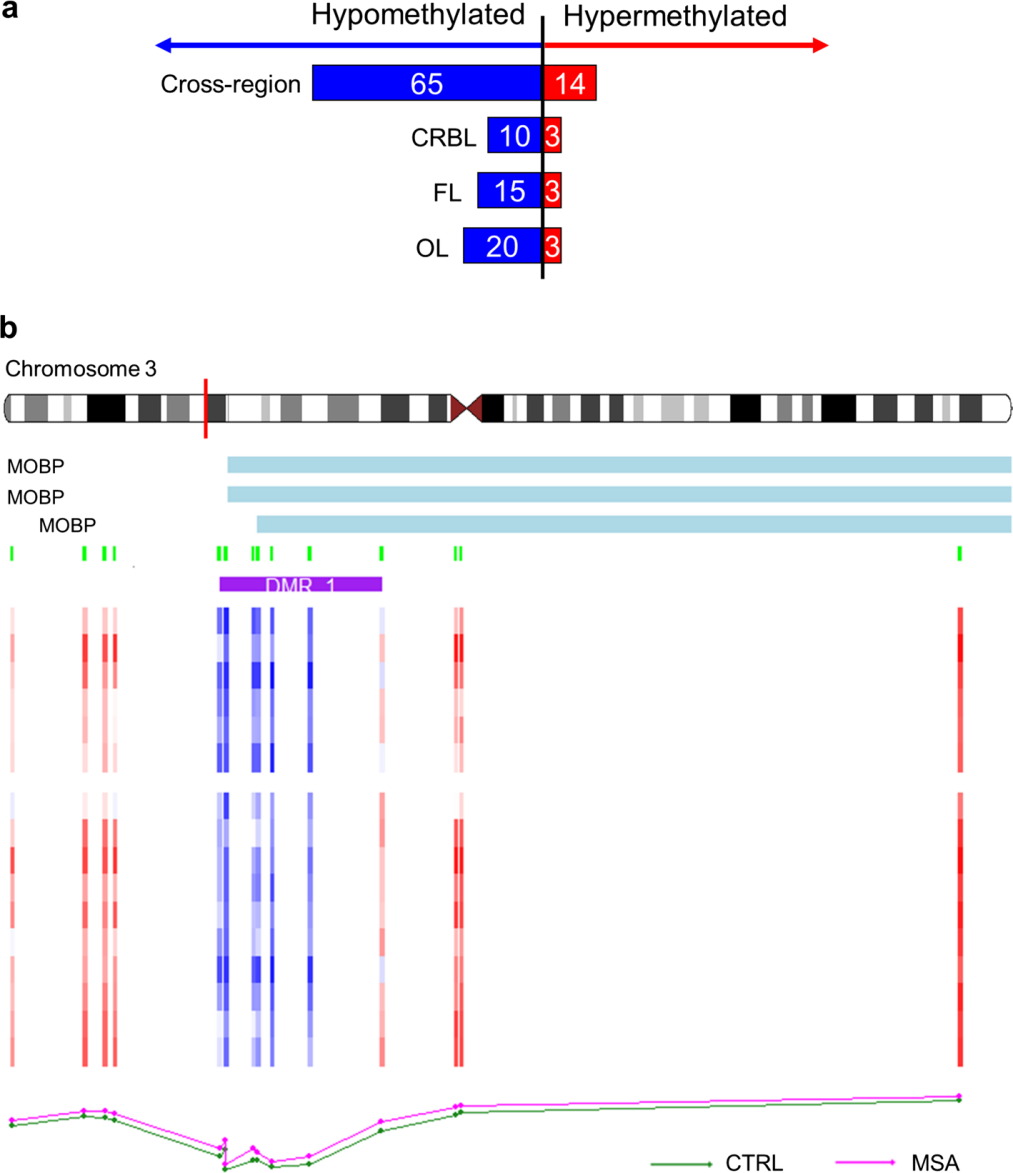
Differentially methylated regions (DMRs) in MSA mixed subtype compared to controls (discovery phase). **a** Number of hypo- and hypermethylated DMRs for the cross-region and region-specific analyses. **b** Top DMR in the cerebellum-specific analysis mapping to *MOBP* on chromosome 3. *CTRL* control group, *MSA* MSA mixed-subtype group, *CRBL* cerebellar white matter, *FL* frontal lobe white matter, *OL* occipital lobe white matter

We next wanted to explore differential methylation spanning larger genomic regions when comparing MSA mixed-subtype cases with controls. In the cross-region analysis (i.e. pooling the three brain regions together), a total of 79 differentially methylated regions (DMRs, Fig. 4a) overlapping with promoter regions of 64 genes were identified. When ranked based on adjusted *p* values, the top DMRs (Supplementary Table S3.1, Online Resource 4) overlapped with the promoters of *PIWIL1* (14 CpGs spanning 1366 bp), *CIZ* (6 CpGs spanning 923 bp), *ITGB2* (12 CpGs spanning 1039 bp), and *MOBP* (8 CpGs spanning 812 bp). Most of the DMRs identified in this cross-region analysis showed loss of methylation in MSA (Fig. 4a), which contrasts drastically with what we found in the analysis of individual CpGs (Fig. 3b), where most sites showed a gain of methylation in MSA. Functional enrichment analysis using these DMRs (64 mapping to gene promoters) revealed a significant overrepresentation of genes related to “piRNA binding” (*p* = 2.00 × 10^−3^) and “polysome binding” (*p* = 4.99 × 10^−3^), suggesting a role in regulation of gene expression and protein production (Supplementary Table S4, Online Resource 5).

Brain region-specific analysis identified 13 DMRs in the cerebellum, 18 in the frontal lobe, and 20 in the occipital lobe (Fig. 4a, Supplementary Tables S3.2–S3.4, Online Resource 4). As for the cross-region analysis described above, the vast majority of DMRs were hypomethylated in MSA compared to controls (Fig. 4a). We detected an overlap across the three brain regions, including the top DMR with the lowest adjusted *p* value in the cerebellum, which maps to *MOBP* (Fig. 4b), and is also in the top significant DMRs for the other brain regions, spanning 7–8 CpGs across 453–812 bp (Supplementary Tables S3.2–S3.4, Online Resource 4). A DMR in *CIZ*, spanning 5–6 CpGs across 550–923 bp, is the second most significant in the cerebellum and is also at the top in the other brain regions (Supplementary Tables S3.2–S3.4, Online Resource 4). Several DMRs were significantly detected in a single brain region, including several DMRs in the cerebellum, which overlap with promoters of *PCSK9*, *RP11-373N24.2*, *NARS*, and *FANCD2OS* (Supplementary Table S3.2, Online Resource 4). As per the analysis of individual CpGs above, most DMRs were shared across brain regions in the MSA mixed subtype, which may be related with more broad systemic changes occurring in MSA. On the other hand, a few DMRs seem to be specific to the cerebellum, which may suggest they are involved in selective vulnerability of this brain region in MSA.

### DNA methylation in *HIP1*, *LMAN2* and other loci is consistently deregulated in MSA mixed-subtype cases

**Fig. 5:**
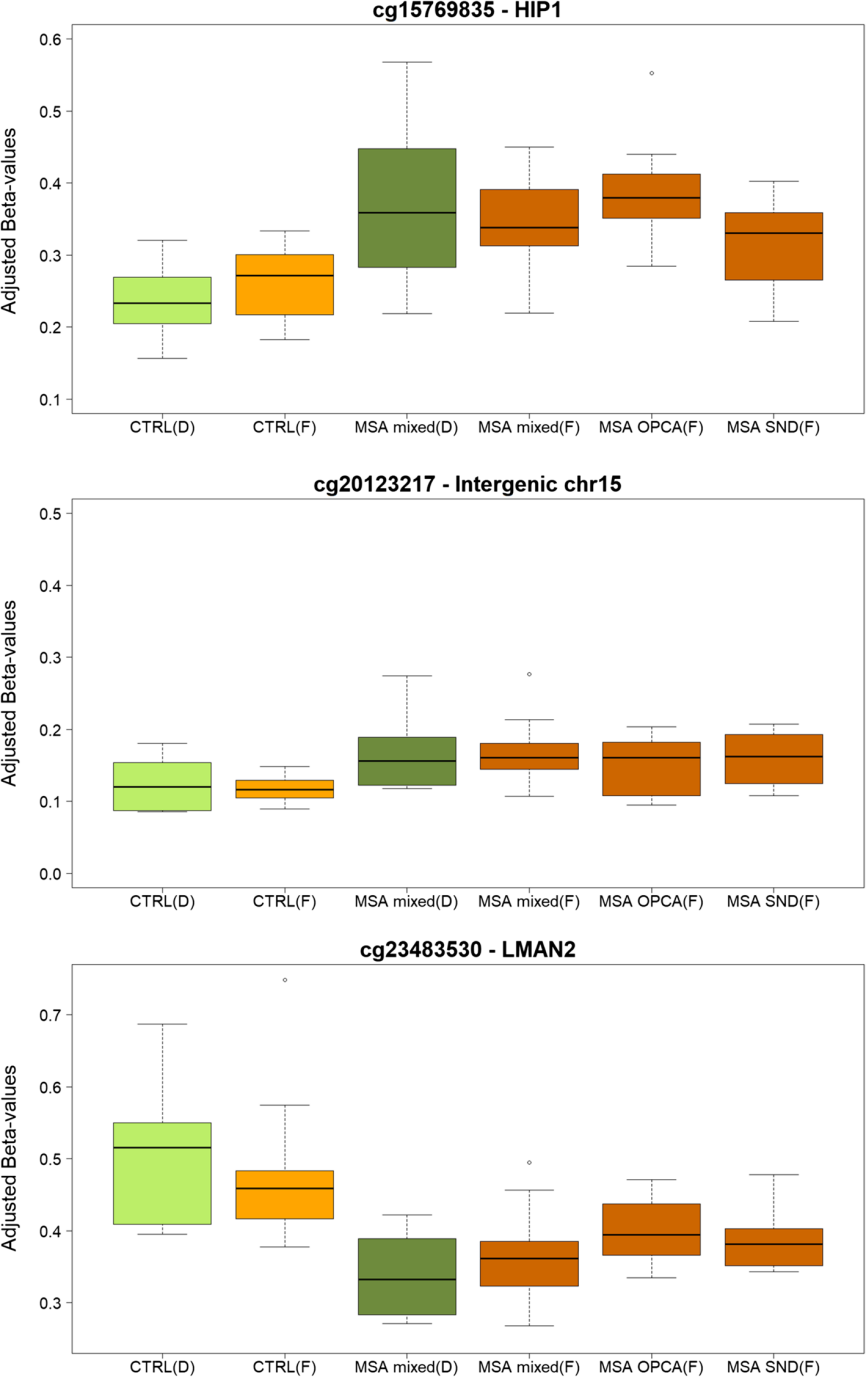
Boxplots of DNA methylation levels in the cerebellar white matter for the top 3 replicated CpGs in the MSA mixed-subtype across all sample groups. Green boxes highlight the discovery cohort (D), and orange boxes highlight the follow-up cohort (F), with light colours corresponding to controls, and dark colours to MSA cases. *CTRL* controls, *MSA* multiple system atrophy, *Mixed* mixed subtype, *OPCA* olivopontocerebellar atrophy subtype, *SND* striatonigral degeneration subtype

Having identified significant alterations to DNA methylation profiles in brain regions affected by MSA, and having shown that these alterations are mostly shared across brain regions (Fig. 3d), we next aimed to replicate these findings in an independent cohort of MSA mixed cases and controls using the cerebellum white matter only. Through stringent quality control steps, 107,166 probes were filtered out from the over 850,000 sites present in the EPIC arrays (for the reasons explained above), and 758,752 CpG sites remained for downstream analysis. Although no CpGs were found to be differentially methylated at the genome-wide level (FDR < 0.05), 11 of the CpGs found to be differentially methylated in the discovery cohort (out of 41 CpGs in the cerebellum and 80 CpGs in the cross-region analyses with absolute delta beta values ≥ 5%, FDR < 0.05) showed a similar direction and magnitude of the effect (absolute delta beta ≥ 5%) in the replication MSA mixed-subtype cohort (Table 1). Although additional CpGs have shown consistent results between the discovery and replication cohorts, we have chosen a minimum absolute delta beta of 5% to ensure the detected differences were robust. A CpG mapping to *HIP1* (cg15769835), an intergenic CpG on chromosome 15 (cg20123217), and another CpG mapping to *LMAN2* (cg23483530) reached nominal significance in the replication cohort of MSA mixed-subtype cases when compared to controls (*p* < 0.05, Fig. 5). It is of note that in terms of effect size (delta betas), these three CpGs were in the top 12 of differentially methylated CpGs in the cross-region analysis of the discovery cohort (Supplementary Table S2.1, Online Resource 2), with absolute delta betas > 5% in all three brain regions. The *LMAN2*-cg23483530 showed significant changes in all three brain regions in the discovery MSA mixed-subtype cohort (FDR < 0.02, delta betas: − 13% cerebellum, − 10% frontal, and − 12% occipital lobe), and even surpassed a conservative Bonferroni significance threshold in the cross-region analysis (*p* = 3.6 × 10^−8^). Following those CpGs, several others also showed very consistent effects in both of the MSA mixed-subtype cohorts compared with controls (Table 1, *p* > 0.05). From the three replicated CpGs, a meta-analysis of all MSA mixed vs all controls confirms a strong effect of cg15769835 (*HIP1*), cg20123217 (intergenic on chromosome 15), and cg23483530 (*LMAN2*), all with delta betas of over 8% and cg20123217 even surpassing a conservative Bonferroni significance threshold (*p* = 1.4 × 10^−9^, Supplementary Table S5, Online Resource 6). *Q*–*Q* plots for the meta-analysis are shown in Supplementary Fig. 1 (Online Resource 3).

**Table 1:** CpGs differentially methylated in the MSA mixed discovery cohort (absolute delta beta ≥ 5% and FDR < 0.05) and maintaining the same direction and magnitude of effect (absolute delta beta ≥ 5%) in the MSA mixed replication cohort

CpG	Delta beta discovery MSA mixed	Delta beta replication MSA mixed	P.Val replication MSA mixed	Delta beta MSA OPCA	P.Val MSA OPCA	Delta beta MSA SND	P.Val MSA SND	Chr	Map (GRCh37)	Gene symbol	Gene name	Feature
cg15769835	**0.15**	**0.08**	**0.01**	**0.31**	**7.94E−04**	**0.08**	0.09	7	75317253	HIP1	Huntingtin interacting protein 1	Body-opensea
cg20123217	**0.10**	**0.10**	**0.02**	**0.18**	**0.02**	**0.11**	**0.01**	15	62418105			IGR-opensea
cg23483530*	** − 0.13**	** − 0.05**	**0.04**	0.01	0.27	** − **0.03	0.12	5	176759977	LMAN2	Lectin, mannose binding 2	Body-shore
cg05199761*	** − 0.10**	** − 0.09**	0.07	** − 0.11**	0.22	0.00	0.68	10	6105145	IL2RA	Interleukin 2 receptor subunit alpha	TSS1500-opensea
cg09418084	**0.08**	**0.06**	0.10	**0.10**	0.98	**0.10**	**0.03**	7	66007964	GS1-124K5.11		Body-opensea
cg10409981*	** − 0.07**	** − 0.06**	0.13	** − 0.06**	0.70	** − 0.05**	0.47	8	99984763			IGR-island
cg14321861*	**0.09**	**0.05**	0.26	0.04	0.39	**0.05**	0.25	17	27287531	SEZ6	Seizure related 6 homolog	ExonBnd-opensea
cg17416644	** − 0.06**	** − 0.07**	0.32	** − 0.28**	0.80	** − 0.10**	0.42	11	1474841	BRSK2	BR serine/threonine kinase 2	Body-shelf
cg21491246	**0.10**	**0.08**	0.37	** − 0.19**	0.67	0.00	0.44	6	15037318			IGR-opensea
cg24955660	**0.18**	**0.06**	0.53	0.00	0.57	0.04	0.55	7	153687910	DPP6	Dipeptidyl peptidase like 6	Body-opensea
cg06209906*	**0.08**	**0.07**	0.54	**0.13**	0.24	0.03	0.47	14	91252201	TTC7B	Tetratricopeptide repeat domain 7B	Body-opensea

*In the discovery cohort these were found to be genome-wide significant in both cerebellum-specific and cross-region analysis (FDR < 0.05), the other CpGs were found to be significant in the cross-region analysis only. Bold highlights CpGs showing absolute delta beta values > 5% and *p* values < 0.05

Next, we wanted to gain depth in understanding the extent of the changes in DNA methylation at the loci identified in the discovery cohort. To do this, using the list of genes showing differentially methylated CpG sites in the MSA mixed-subtype discovery cohort, we investigated whether DNA methylation levels in the cerebellum at additional CpG sites in these same genes were significantly affected in the MSA mixed-subtype replication cohort. We identified 10 CpGs showing gene-wide significant changes in the MSA mixed-subtype replication cohort compared to controls with a consistent direction of effect compared to the discovery cohort (Table 2a). Of note, three of these additional CpGs were in the *DGKI* gene, a gene that has shown cerebellum-specific DNA methylation changes in the discovery cohort. We also asked whether there would be significant DNA methylation changes at CpG sites spanning differentially methylated regions found in the discovery phase (significance threshold *p* < 7.81 × 10^−4^ [0.05/64 gene promoters]). Only one CpG site in *MDGA1* (cg20053110), found to be significantly hypomethylated in the MSA mixed-subtype replication cohort compared to controls (delta beta = − 12%, *p* = 3.50 × 10^−4^), overlaps with a 260-bp region (4 CpG sites) significantly hypomethylated in the discovery cohort.

**Table 2:** Genes with at least one differentially methylated CpG in the discovery cohort (absolute delta-beta ≥ 5%, adj. *p* < 0.05) and with additional CpGs differentially methylated in the MSA mixed replication cohort and/or MSA OPCA (absolute delta-beta ≥ 5%, gene-wide significant)

CpG	Delta beta discovery MSA mixed	Delta beta replication MSA mixed	P.Val replication MSA mixed	Delta beta MSA OPCA	P.Val MSA OPCA	Delta beta MSA SND	P.Val MSA SND	Chr	Map (GRCh37)	Gene symbol	Gene name	Feature
(a) Gene-wide significant changes in the MSA mixed replication cohort and occasionally in MSA OPCA [cross-region significance threshold *p* < 9.00E−04 (0.05/54 genes) or cerebellum-specific threshold *p* < 1.90E − 03 (0.05/27 genes)]												
cg15271829***	** − **0.03	** − 0.06**	**4.04E−04**	** − 0.22**	**7.91E−05**	** − **0.04	5.40E−01	2	28789262	PLB1	Phospholipase B1	Body-shore
cg08313420*	** − **0.01	** − 0.07**	**8.90E−04**	** − 0.25**	3.37E−03	** − 0.08**	2.32E−02	7	6476110	DAGLB	Diacylglycerol lipase beta	Body-opensea
cg00282245*	**0.05**	**0.09**	**5.27E−04**	** − 0.06**	4.74E−02	0.01	2.36E−01	7	51130590	COBL	Cordon-bleu WH2 repeat protein	Body-opensea
cg14212951***	** − **0.02	** − 0.07**	**3.32E−04**	0.03	7.90E−02	** − **0.03	3.64E−02	7	137139878	DGKI	Diacylglycerol kinase iota	Body-opensea
cg05540100***	** − **0.03	** − 0.12**	**5.38E−06**	** − **0.0003	**1.72E−03**	** − **0.02	2.39E−02	7	137367050	DGKI	Diacylglycerol kinase iota	Body-opensea
cg02113214***	** − **0.01	** − 0.06**	**9.34E−05**	** − **0.01	**1.84E−03**	** − **0.02	4.78E−02	7	137410625	DGKI	diacylglycerol kinase iota	Body-opensea
cg10264318*	** − **0.02	** − 0.07**	**5.93E−04**	**0.05**	4.09E−01	** − **0.02	1.35E−01	7	154633168	DPP6	Dipeptidyl peptidase like 6	Body-opensea
cg12499572***	**0.06**	**0.09**	**8.26E−04**	**0.28**	**7.62E−05**	**0.07**	3.52E−01	10	134462831	INPP5A	Inositol polyphosphate-5-phosphatase A	Body-shelf
cg22343263*	** − **0.03	** − 0.05**	**1.00E−04**	0.002	3.48E−02	** − **0.001	1.06E−01	12	50466510	ASIC1	Acid sensing ion channel subunit 1	TSS1500-opensea
cg18498263***	0.03	**0.05**	**1.19E−03**	** − 0.06**	8.36E−03	0.002	1.55E−01	14	91034963	TTC7B	Tetratricopeptide repeat domain 7B	Body-opensea
(b) Gene-wide significant in OPCA only [cross-region significance threshold *p* < 9.00E−04 (0.05/54 genes) or cerebellum-specific threshold *p* < 1.90E−03 (0.05/27genes)]												
cg16729794*	**0.06**	**0.09**	0.016	**0.28**	**1.83E−05**	**0.08**	2.22E−01	3	39509195	MOBP	Myelin-associated oligodendrocyte basic protein	5′UTR-opensea
cg24602838*	**0.09**	**0.10**	0.003	**0.33**	**3.86E−04**	**0.08**	4.90E−01	3	39527173	MOBP	Myelin-associated oligodendrocyte basic protein	5′UTR-opensea
cg05317077*	**0.08**	**0.08**	1.34E−02	**0.26**	**7.22E−04**	**0.09**	1.48E−01	3	39542991	MOBP	Myelin-associated oligodendrocyte basic Protein	TSS1500-shore
cg06277657***	** − **0.03	** − **0.01	0.318	** − 0.08**	**1.06E−03**	** − **0.03	5.78E−02	7	137532374	DGKI	Diacylglycerol kinase iota	TSS1500-island
cg10947146**	** − **0.02	** − **0.02	0.006	** − 0.06**	**2.73E−04**	** − **0.02	2.94E−02	8	11058710	XKR6	XK related 6	1st exon-island
cg02881186***	** − **0.004	** − **0.02	0.012	** − 0.22**	**6.51E−05**	** − **0.03	4.17E−01	8	140892009	TRAPPC9	Trafficking protein particle complex 9	Body-opensea
cg10445361***	**0.08**	**0.06**	0.027	**0.24**	**1.05E−03**	**0.05**	5.85E−01	10	134461611	INPP5A	Inositol polyphosphate-5-phosphatase A	Body-shore
cg13582959***	**0.05**	**0.08**	0.004	**0.26**	**4.28E−04**	**0.05**	9.95E−01	10	134462849	INPP5A	Inositol polyphosphate-5-phosphatase A	Body-shelf
cg02240936***	**0.05**	0.04	0.687	**0.09**	**1.53E−03**	0.02	8.32E−01	10	134584660	INPP5A	Inositol polyphosphate-5-phosphatase A	Body-shore
cg03237845***	**0.11**	**0.08**	0.033	**0.25**	**4.13E−04**	**0.05**	9.36E−01	15	57377660	TCF12	Transcription factor 12	Body-opensea

*Gene with significant CpG(s) in the cross-region analysis only; **gene with significant CpG(s) in cerebellum-specific analysis only; ***with significant CpG(s) in both cerebellum-specific and cross-region analyses; bold highlights CpGs showing absolute delta beta values > 5% and gene-wide significant *p* values

### The intensity of cerebellar DNA methylation deregulation varies between MSA pathological subtypes

To explore the relationship between DNA methylation changes and MSA pathology burden further, we have investigated the MSA OPCA subtype in which, similarly to the MSA mixed-subtype, the cerebellum is severely affected, as well as the MSA SND subtype, with less severe involvement of the cerebellum compared to striatonigral regions. We found that out of the 11 CpGs consistently showing changes across MSA mixed-subtype cohorts (Table 1, Fig. 5), 7 (63.6%) exhibit the same direction and magnitude of effect in MSA OPCA, and 6 (54.5%) in MSA SND. The CpG in *HIP1* (cg15769835) showed a strikingly stronger and significant effect in MSA OPCA subtype (delta beta > 30%, Table 1). The intergenic CpG-cg20123217 reached nominal significance in all cohorts, and showed again a stronger effect in MSA OPCA (Table 1). On the other hand, the CpG in *LMAN2* (cg23483530) does not show the same trend in MSA OPCA compared to the other MSA subtypes. For MSA SND, apart from the intergenic CpG-cg20123217, the cg09418084 mapping to a pseudogene also reached nominal significance (Table 1).

Using the list of genes showing differentially methylated CpG sites in the MSA mixed discovery subtype cohort we investigated whether additional CpG sites in these same genes were significantly altered in the MSA OPCA and MSA SND subtypes. Four out of ten CpGs showing gene-wide significant changes in the MSA mixed-subtype replication cohort compared to controls were also significant in MSA OPCA (Table 2a). Two of these CpGs map to *DGKI* and exhibited very small changes (less than 2%), but the other two CpGs (cg15271829-*PLB1* and cg12499572-*INPP5A*, Table 2a) showed much stronger effects in MSA OPCA compared to the other MSA subtypes. Several other CpGs presented consistent effects in all cohorts but much stronger and significant changes in MSA OPCA only (Table 2b). These include multiple changes in the same genes (another CpG in *DGKI*, three in *INPP5A*, and two in *MOBP*). It is of note that in the discovery cohort a CpG in *INPP5A* was the top differentially methylated CpG in both the cross-region and region-specific analyses (second in occipital lobe only), a CpG in *DGKI* was found to be significant in the cerebellum only, and also the top differentially methylated region in the cerebellum (also detected in the other brain regions) overlapped with the *MOBP* promoter region.

Regarding CpGs located around the DMRs found in the discovery phase, only three CpG sites in *MOBP* (cg16729794, cg24602838, and cg05317077), located just upstream of the 812-bp region (8 CpG sites) which corresponded to the top DMR in the cerebellum and was hypermethylated in all three brain, were found to be significantly hypermethylated in MSA OPCA (delta betas > 26%, Table 2) and have shown the same trend but with smaller effects in MSA mixed and MSA SND subtypes (delta betas = 6–10%, Table 2).

### Cerebellar co-methylation networks identify DNA methylation signatures associated with MSA disease status

Next, to provide insight into higher order relationships across CpGs/genes, we have constructed co-methylation networks using the top 10% most variable CpGs in all cerebellar samples. Using this agnostic systems biology approach based on WGCNA, we have identified 45 clusters of highly correlated CpGs, called co-methylation modules from now on and each assigned a colour name.

**Fig. 6:**
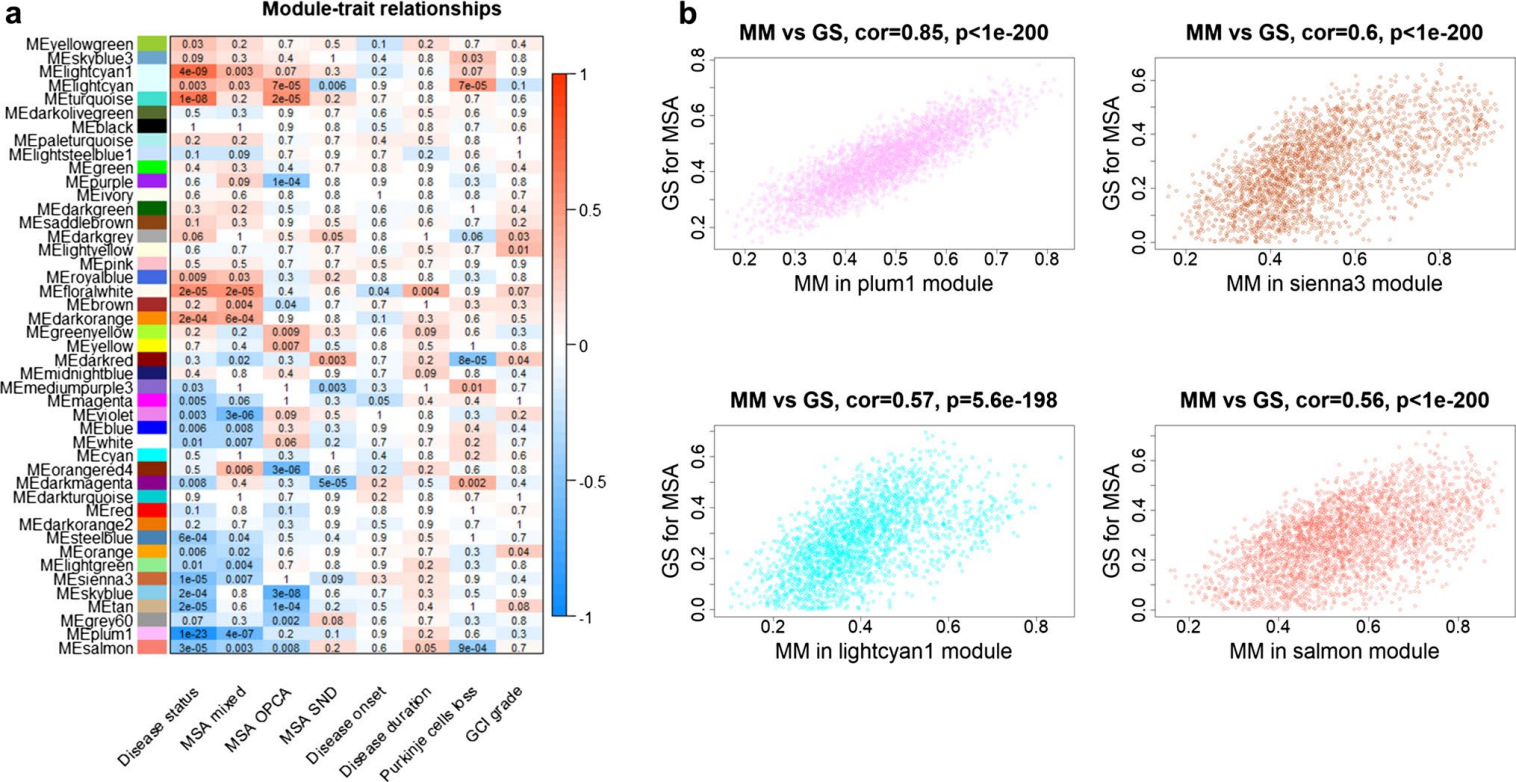
Results from the cerebellar multiple system atrophy (MSA) co-methylation network analysis. **a** Module–trait relationships. The rows represent the co-methylation module eigengene (ME) and its colour, and the columns represent clinical/pathological traits. *P* values are presented within each cell and the colour scale at the right indicates the strength of the correlation (darker cells depict stronger correlations, with blue representing negative and red positive correlations). **b** Correlations between module membership (MM) and gene significance (GS) for MSA for the top 4 modules associated with the MSA status (*r* > 0.50). *MSA* multiple system atrophy, *Mixed* mixed subtype, *OPCA* olivopontocerebellar atrophy subtype, *SND* striatonigral degeneration subtype, *GCI* glial cytoplasmic inclusions

Ten out of the 45 identified co-methylation modules (plum1, lightcyan1, turquoise, sienna3, floralwhite, tan, salmon, darkorange, steelblue, and skyblue, Fig. 6a) were found to be associated with the disease status (i.e. MSA or control) regardless of the MSA pathological subtype (*p* < 0.001, 0.05/45 modules). The module membership (MM) values for CpGs in MSA-associated modules were highly correlated with gene significance (GS) for MSA, suggesting that the CpGs with the highest MM (hub CpGs, Table 3) within those modules are the most relevant for MSA. The top 4 modules showing the stronger correlations between MM and GS (*r* > 0.5) are represented in Fig. 6b, and will be further described below.

**Table 3:** Highly interconnected CpGs in co-methylation modules associated with the MSA disease status

Module	CpGs	Genes	Chr	Feature	Module	CpGs	Genes	Chr	Feature
Plum1^a^	cg20048521	C6orf221	6	TSS200	Tan^b^	cg06642177	SGK1	6	Body
	cg14428827		7	IGR		cg14078059		12	IGR
	cg18932707		6	IGR		cg11162385	NANP	20	TSS200
	cg09303171	ITGA8	10	Body		cg15238008	DDX12	12	TSS200
	cg25842050		5	IGR		cg26460378	WDR62	19	TSS200
	cg16201233	RRAGC	1	Body		cg16508039	UBR5-AS1	8	TSS1500
	cg06148118	LMO2	11	TSS1500		cg02653800	UBE2D1	10	Body
	cg07945047		7	IGR		cg13113052	POLI	18	Body
	cg01495416		8	IGR		cg20882291	SDC2	8	5′UTR
	cg26195291	EPB41L2	6	5′UTR		cg14714046		6	IGR
Lightcyan1	cg06478421		2	IGR	Salmon	cg01095763	IQCA1	2	Body
	cg16787431	HOXB3	17	Body		cg24397470	FRMD4A	10	Body
	cg20446143	MS4A8B	11	TSS1500		cg16905100		3	IGR
	cg10426234		12	IGR		cg07191498	DTNA	18	5′UTR
	cg08380642	SLC9A5	16	Body		cg11919558	SERTAD2	2	5′UTR
	cg18624016	SNED1	2	Body		cg05852537	PROCR	20	TSS200
	cg25989511		5	IGR		cg24003508	KLF6	10	Body
	cg22344631		2	IGR		cg18284089	TMEM154	4	TSS200
	cg02707071	HOXA2	7	TSS1500		cg03353442	DENND4C	9	5′UTR
	cg13527466		12	IGR		cg04337518	AMPD2	1	TSS1500
Turquoise^b^	cg20051635	ZAP70	2	ExonBnd	Darkorange^a^	cg06122635		2	IGR
	cg07600621		1	IGR		cg08769544	MEPCE	7	TSS1500
	cg21588562	FTMT	5	TSS200		cg07667367	SPATA4	4	TSS200
	cg16901862		12	IGR		cg03309025		3	IGR
	cg23523282		19	IGR		cg07287188		17	IGR
	cg07524919	TNXB	6	Body		cg25730577	CSF1	1	TSS1500
	cg10365886	TNXB	6	Body		cg00867446	ACIN1	14	5′UTR
	cg07945971	TSPAN9	12	5′UTR		cg22506042	P2RX1	17	Body
	cg00872984	TNXB	6	Body		cg26538214	KLF6	10	5′UTR
	cg10923662	TNXB	6	Body		cg05326918	VPS4A	16	TSS1500
Sienna3	cg21051046		7	IGR	Steelblue	cg05966983	C1orf113	1	TSS200
	cg12371933		7	IGR		cg12071656	MMP9	20	Body
	cg19112895		3	IGR		cg06213463	MT3	16	TSS200
	cg14349532		3	IGR		cg00351804		7	IGR
	cg18966597		7	IGR		cg13609723	NKX3-1	8	Body
	cg12105980		7	IGR		cg07137043	EIF4H	7	Body
	cg06597462		7	IGR		cg07008893	CHD7	8	5′UTR
	cg16929199		7	IGR		cg26474620	ST7	7	Body
	cg11382477		7	IGR		cg19812670	NAALADL2	3	Body
	cg13482308	PAX5	9	Body		cg05225581	KBTBD6	13	1stExon
Floralwhite^a^	cg21734015	FOXA3	19	TSS1500	Skyblue^b^	cg17398237	MND1	4	TSS200
	cg04599533	LMLN	3	Body		cg23904393	NCBP1	9	5′UTR
	cg25471021	MTA3	2	Body		cg17430625	MPV17L2	19	Body
	cg10434152	GOLSYN	8	TSS200		cg03594908		7	IGR
	cg25936334	CCDC82	11	5′UTR		cg22717608	PDHX	11	TSS200
	cg09170127	ZMAT2	5	Body		cg08656770		6	IGR
	cg13527872	DAPK1	9	Body		cg16886471		17	IGR
	cg13064050	GPR37	7	TSS200		cg00116554	SNAI2	8	1stExon
	cg17819963	PAOX	10	TSS1500		cg18721249	CAV1	7	Body
	cg19731597	NCAN	19	TSS200		cg00013410	MED13	17	1stExon

^a^Also associated with the MSA mixed subtype

^b^Also associated with the MSA OPCA subtype

One module (plum1) presented a strikingly strong negative correlation with the disease status (*r* = − 0.91, Fig. 6, Table 3), suggesting lower levels of methylation in this cluster of CpGs in MSA compared to controls. It is of note that this module contains a CpG in *SNCA* (cg15402943), and also a CpG in *DGKI* (cg05540100) found to be significantly hypomethylated in MSA compared to controls in our previous analysis (Table 2). The top enriched pathway for this module is “Cell death signalling via NRAGE, NRIF and NADE”, followed by “Rho GTPase cycle”, “Diseases of signal transduction”, “Axon guidance”, and “Splicing factor NOVA regulated synaptic proteins” (Supplementary Table S6.1, Online Resource 7). The module (sienna3) presenting the second strongest negative association with MSA (*r* = − 0.54, Fig. 6, Table 3), contains a CpG in *LMAN2* (cg23483530) found to be differentially hypomethylated in MSA mixed subtype (Table 1). Most of the hub CpGs in this module are intergenic (Table 3). It is of note that this module is enriched for “Cerebral white matter atrophy” (Human phenotype ontology term), and for infection related pathways (Supplementary Table S6.2, Online Resource 7). Another interesting module is the lightcyan1 module (*r* = 0.68, Fig. 6, Table 3). Elevated DNA methylation in the *HOXA* gene cluster on chromosome 7 has been associated with AD neuropathology [45], which is in line with the positive correlation found between the lightcyan1 module (containing *HOXA2* and additional CpGs in this region of chromosome 7, Table 3) and the MSA status. The lightcyan1 module is enriched for drug metabolism related pathways (Supplementary Table S6.3, Online Resource 7). A fourth interesting module is the salmon module (*r* = − 0.52, Fig. 6, Table 3), which has an overrepresentation of CpGs mapping to genes involved in “Axon guidance”, and “Rho GTPase cycle” (Supplementary Table S6.4, Online Resource 7). Functional gene ontology analysis across these four modules shows a shared enrichment for “cytoskeletal protein binding”, “anatomical structure development”, “cell morphogenesis”, “nervous system development”, and “cell projection”. Detailed results on the functional enrichment analysis for all modules associated with the MSA status is provided in Supplementary Tables S6.1–6.10 (Online Resource 7).

### Co-methylation network analysis identifies MSA subtype-specific signatures in the cerebellum

Our co-methylation network analysis not only revealed signatures associated with the MSA disease status but also co-methylation modules associated specifically with each pathological MSA subtype (Fig. 6a). Although there is overlap across MSA subtypes in terms of directionality (positive or negative correlations) with several modules, modules reaching network-wide significant correlations (*p* < 0.001) are unique to each MSA subtype (Fig. 6a). The most highly interconnected CpGs (hub CpGs) for each of the MSA subtype-associated modules are shown in Tables 3 and 4.

**Table 4:** Highly interconnected CpGs in co-methylation modules exclusively associated with one of the MSA pathological subtypes

Module	CpGs	Genes	Chr	Feature	Association
Violet	cg00566635	ADAMTS8	11	5′UTR	MSA mixed
	cg07058484		16	IGR	
	cg20697767	RPRML	17	1stExon	
	cg09728904	ASS1	9	Body	
	cg07049764		6	IGR	
	cg01040624	SLC12A8	3	Body	
	cg21242212	MIR208B	14	TSS1500	
	cg08388822	ZSWIM7	17	Body	
	cg08318600	C16orf38	16	Body	
	cg05788548	SKI	1	Body	
Lightcyan	cg10341355	SLC13A3	20	Body	MSA OPCA
	cg00837537		8	IGR	
	cg12408507	DYSF	2	Body	
	cg01481205	MIR4534	22	TSS1500	
	cg00213091	INPP5A	10	3′UTR	
	cg19851810	EPHB2	1	Body	
	cg04315300		1	IGR	
	cg09033376	ASPRV1	2	1stExon	
	cg12945363	RGS3	9	Body	
	cg11931953	FOXK1	7	Body	
Purple	cg04976746	SLC30A1	1	Body	MSA OPCA
	cg14244136	TPM1	15	1stExon	
	cg10088041	LOC145663	15	TSS200	
	cg01688355	FAR1	11	5′UTR	
	cg10654015	TP73	1	TSS200	
	cg04346539	ORMDL2	12	TSS1500	
	cg03174294	GPR124	8	5′UTR	
	cg14042203	DTX4	11	TSS200	
	cg26100711	MIXL1	1	TSS200	
	cg11142248	FLJ43390	14	Body	
Orangered4	cg12059180	CLDN18	3	TSS1500	MSA OPCA
	cg20506380	SPTA1	1	Body	
	cg06797478		13	IGR	
	cg11977158	SYN3	22	Body	
	cg11275803	RPS18	6	TSS1500	
	cg12456435	CSDE1	1	5′UTR	
	cg27052442	ERBB2	17	5′UTR	
	cg12436715	TTLL3	3	3′UTR	
	cg22966196	ABCC2	10	Body	
	cg16039979	C1orf86	1	5′UTR	
Darkmagenta	cg22674699	HOXD9	2	1stExon	MSA SND
	cg02792822	PSAP	10	Body	
	cg14141984	SETD1A	16	Body	
	cg15768526	KNOP1	16	5′UTR	
	cg27151617	ANKRD7	7	TSS1500	
	cg25015867	ZNF521	18	Body	
	cg05386151	NUCKS1	1	5′UTR	
	cg05906350	FAM190B	10	5′UTR	
	cg07959065	AMFR	16	Body	
	cg03816598		2	IGR	

Four modules are associated with the MSA mixed subtype (floralwhite, darkorange, violet, and plum1, *p* < 0.001; Fig. 6a, Tables 3, 4). As per the analysis with the overall MSA status, the module presenting the strongest association with MSA mixed subtype is plum1, which has been described above. These four modules are enriched for “cytoplasm”, and “intracellular organelle part” as well as for a transcription factor binding motif for *E2F* (Supplementary Tables S6.1, S6.6, S6.8, and S6.11, Online Resource 7). Furthermore, with the exception of the violet module, the other three modules present an enrichment for “enzyme binding” and “protein-containing complex” as well as for several shared transcription factors binding motifs. Regarding molecular pathways, the floralwhite module is enriched for “Mitophagy—animal”.

Six modules are associated with the MSA OPCA subtype (lightcyan, turquoise, purple, orangered4, skyblue, and tan, *p* < 0.001; Fig. 6a, Tables 3, 4). The lightcyan module is positively correlated with MSA OPCA and contains the *HIP1* CpG (cg15769835), which was found to be differentially hypermethylated in the MSA mixed and the MSA OPCA subtypes, with a much stronger effect in the latter (Table 1). This CpG in *HIP1* has the 6th highest gene significance for MSA in the lightcyan module. It is of note that one of the hub CpGs of the lightcyan module maps to *INPP5A*, which was also detected in our differential methylation analysis, both in the MSA mixed discovery cohort and in the MSA OPCA subtype (Table 2; Supplementary Tables S1.1–1.4, Online Resource 1). Also of note, and in line with what we observed in the differential methylation analysis, the sienna3 module containing the *LMAN2* CpG (cg23483530) is not associated with MSA OPCA (*r* = − 0.0025, *p* = 0.985). These modules are enriched for “protein binding” and “neurogenesis”, “cytoplasm”, and “organelle part” as well as for several shared transcription factors binding motifs (4/6 modules). Regarding the enrichment for molecular pathways, the purple and tan modules are enriched for “Ubiquitin mediated proteolysis”, and the skyblue and tan are enriched for “FoxO signalling pathway” and “Gene expression (Transcription)”. The lightcyan module is enriched for “Axon guidance”, while the turquoise is enriched for “Long-term depression”, and the orangered4 for “Synaptic vesicle cycle” (Supplementary Tables S6.5, and S6.12–6.14, Online Resource 7).

Since the cerebellum is mildly affected in MSA SND subtype, we were expecting to find fewer modules associated with this MSA subtype in cerebellar networks. Indeed, only one module was found to be associated with MSA SND (darkmagenta; Fig. 6a, Table 4), which is enriched for “protein domain specific binding” (Supplementary Table S6.15, Online Resource 7).

### Co-methylation network analysis identifies MSA neurodegeneration-associated signatures in the cerebellum

To further investigate the relationship between methylation signatures and disease features, we have also correlated the co-methylation modules with some of the major disease related traits, including disease onset, disease duration, Purkinje cell loss (as a proxy for neurodegeneration) and GCI grade/burden. We found three modules significantly associated with Purkinje cell loss in the cerebellum (lightcyan, darkred, and salmon, *p* < 0.001; Fig. 6a), but no other significant associations with the other disease traits after accounting for multiple testing. The lightcyan module (positively associated with the MSA OPCA subtype, and including the CpG in *HIP1*) shows a positive correlation with loss of Purkinje cells in the cerebellum (Fig. 6a). This finding suggests that higher methylation levels in the CpGs composing that cluster are associated with increased cell loss. On the other hand, two modules—the darkred (positively correlated with the MSA SND subtype) and the salmon module (negatively associated with the MSA status) show negative correlations with loss of Purkinje cells (Fig. 6a), and therefore higher methylation levels in those CpGs seem to protect against neuronal cell loss. These results suggest that white matter DNA methylation signatures are associated with neurodegeneration in MSA.

## Discussion

To the best of our knowledge, this is the first epigenome-wide study in MSA. We have quantitatively interrogated over 850,000 methylation sites across the genome, covering 99% of RefSeq genes, 95% of CpG islands, and a high number of enhancer regions. Our case–control study was carefully designed (Fig. 1) and aimed first at better understanding the variation in pathological burden in multiple brain regions (cerebellum, frontal lobe and occipital lobe). We also further extended our research to the three main MSA pathological subtypes (MSA mixed, MSA OPCA, and MSA SND). Our discovery cohort identified over 60 differentially methylated CpG sites in each brain region (region-specific analyses), and over 150 CpG sites in a more powerful cross-region analysis. Similar studies performed in other neurodegenerative diseases (e.g. AD), have reported gains in DNA methylation [45]. In line with these reports, our data also show that the vast majority of differentially methylated CpGs in MSA show a gain in DNA methylation. We refer further below to several neurodegeneration-related genes that have shown consistent differential methylation in brain regions affected by MSA across cohorts.

The *HIP1* gene encodes a cytosolic protein (huntingtin interacting protein 1—HIP1), which is ubiquitously expressed and highly enriched in human and mouse brain tissue [51]. The real function of HIP1 protein remains unknown, but it has been shown to have a role in the clathrin-mediated endocytosis, which regulates several signalling pathways, receptor trafficking and cytoskeleton dynamics [39, 50]. HIP1 has been shown to be a direct interactor of huntingtin (the causal protein mutated in Huntington’s disease). In Huntington’s disease, HIP1’s pro-apoptotic activity has been proposed to play a role in the amplification of the cascade of cell death signals [13]. The significant increase in DNA methylation levels in the body of the *HIP1* gene (cg15769835) we observed in MSA overall, and which was more pronounced in MSA OPCA when compared to controls, suggest that HIP1 may have a toxic effect in MSA controlled, at least in part, by DNA methylation.

The closest coding gene upstream of the intergenic CpG on chromosome 15 (cg20123217, with increased DNA methylation levels in MSA) is *VPS13C*, which belongs to a family of large VPS13 proteins (VPS13A–D, vacuolar sorting proteins crucial for vesicular transport) and has been associated with early onset autosomal recessive PD [27]. Interestingly, another intergenic CpG (cg10409981) showing consistent reduction in DNA methylation levels across all MSA cohorts/subtypes, is located upstream of an additional gene in the same family—*VPS13B*, which has been associated with Cohen syndrome, a developmental disorder with intellectual disability, among other central nervous system features [20].

The *LMAN2* gene encodes the vesicular integral-membrane protein VIP36, which is an intracellular lectin that cycles between the endoplasmic reticulum (ER) and the Golgi apparatus, and has been suggested to act as a cargo receptor in the transport and sorting of glycoproteins. Cargo receptors participate in the export of folded proteins from the ER and also in the retrieval of misfolded proteins from the Golgi to the ER [31]. A common feature across neurodegenerative diseases is the accumulation of misfolded proteins, such as α-synuclein in MSA. Dysregulation of *LMAN2* through DNA methylation changes, as the observed reduction of DNA methylation levels in the body of the gene (cg23483530), may be contributing to impaired protein quality control in MSA.

Apart from significant changes in individual CpGs, we also identified differentially methylated regions comprising several CpGs in the MSA mixed subtype (discovery phase). These regions included CpGs mapping to the *MOBP* promoter, which is the top differentially methylated region in the cerebellum. Notably, additional CpGs in *MOBP* were significantly associated with the MSA OPCA subtype in the follow-up phase of our study. The *MOBP* gene encodes the myelin-associated oligodendrocyte basic protein (MOBP), which is the third most abundant protein in CNS myelin [29]. Although the MOBP function remains unclear, it has been suggested that it primarily plays a role in connecting myelin to a membrane-associated signalling complex linked to the cytoskeleton, thus participating in myelin stabilization. Variable levels of MOBP have been shown to affect the morphological differentiation of oligodendrocytes [43], and could therefore be playing a role in increasing the density of oligodendrocyte precursor cells as observed in MSA [1, 10]. Further supporting an involvement of *MOBP* in neurodegenerative diseases, several reports have provided evidence for a role of MOBP in the pathogenesis of multiple sclerosis [29], and genetic variants in *MOBP* have been associated with increased risk of neurodegenerative diseases, including progressive supranuclear palsy (PSP) and corticobasal degeneration [16, 21].

The power of genome-wide DNA methylation studies, such as this one, lies on the fact that they can additionally identify pathways and networks, which may be more relevant to disease pathophysiology than lists of individual genes. It has been suggested that co-methylation, i.e. highly correlated methylation levels across samples, can be used as a proxy to discover functional associations between gene pairs [2]. We have therefore performed a co-methylation network analysis and identified modules associated with the MSA disease status and MSA pathological subtypes. Importantly, within the co-methylation module which displayed the strongest association with MSA (plum1 module) we found a CpG in *SNCA* (cg15402943), the gene that encodes α-synuclein. MSA and PD are α-synucleinopathies, both sharing the accumulation of α-synuclein in inclusions but predominantly in different cell types. In PD, a significant reduction in DNA methylation levels at the *SNCA* promoter region has been reported [18, 48]. Here, in MSA, we found the association with the plum1 module, even though significant changes in individual CpGs mapping to the *SNCA* region were not detected. This suggests that perhaps in MSA *SNCA* plays a role in pathways relevant to the disease but it is not the sole or primary driver of such pathways. In line with reports of apoptotic cell death in MSA, almost exclusively in oligodendrocytes [17, 38], the top enriched pathway for the plum1 module was “Cell death signalling via NRAGE, NRIF and NADE”. This module also contains an overrepresentation of genes involved in the “Rho GTPase cycle”. Rho GTPases are small G proteins, which have been implicated in the pathogenesis of neurodegenerative diseases, such as PD and AD. Rho GTPases are evolutionarily conserved regulators of cytoskeletal dynamics, and function in signalling pathways from the earliest stages of embryonic development and supporting life thereafter [7, 47]. The co-methylation network analysis also revealed other pathways in MSA, including mitophagy and infection related pathways. Mitochondrial dysfunction has indeed been observed in MSA, and has been suggested as a shared disease mechanism among α-synucleinopathies [11]. Furthermore, the overrepresentation of changes in infection related pathways may point to neuroinflammation, another shared mechanism across neurodegenerative diseases [14, 19, 32]. Interestingly, a recent study reports on the crosstalk between neuroinflammation and oligodendrocytes containing GCIs leading to an immune response locally restricted to white matter regions in MSA [15]. The co-methylation network analysis supported and complemented the results from the differential methylation analysis, as MSA-associated modules included CpGs that had been found to be differentially methylated in MSA. These include the CpG in *LMAN2* (cg23483530) in the sienna3 module, which presents the second strongest negative association with the MSA status, and the CpG in *HIP1* (cg15769835) in the lightcyan module, which was positively associated with the MSA OPCA subtype.

As in MSA the oligodendrocytes are the cells most frequently affected by α-synuclein aggregation, it is interesting to note that our findings point to several genes that are mainly expressed in oligodendrocytes, such as *HIP1* and *MOBP*, while *SNCA* is expressed in neurons and oligodendrocytes in similar proportions, and *LMAN2* is more highly expressed in astrocytes and microglia (as seen in single cell RNAseq data, https://www.brainrnaseq.org/). Elucidating the correlation between DNA methylation at these loci and gene expression levels is certainly of interest and warrants future investigation.

There has been an emerging role for axon–oligodendrocyte coupling particularly in MSA, and also in other neurodegenerative diseases [30]. Oligodendrocytes represent the majority of glial cells in the adult CNS (75%). The main and best known function of oligodendrocytes is the production, stability and maintenance of myelin, the organized and tightly packed enlarged plasma membrane of oligodendrocytes that wraps around neuronal axons [10]. Supporting the hypothesis that this function is impaired in MSA, myelin staining is focally reduced in MSA, and this is accompanied by myelin degradation [1]. Also, our data identified DNA methylation changes in the gene encoding the third most abundant myelin protein (MOBP). Other functions of oligodendrocytes include: (a) the maintenance of ionic homeostasis in the CNS, by buffering increased extracellular potassium resulting from neuronal excitation; (b) the provision of metabolic and trophic supply at the axon–myelin unit, helping the maintenance of neuronal functions; (c) the provision of lactate as a source of energy, which is crucial for axonal survival; and (d) the secretion of growth factors, including glial- and brain-derived neurotrophic factor (GDNF and BDNF), which control neuronal survival and axonal outgrowth [10]. Our data suggest that the latter may be affected in MSA, as “Axon guidance” pathway is enriched across several MSA-associated co-methylation modules. It has been shown that oligodendrocyte ablation severely affects cerebellar neuronal circuitries [8]. We have found cerebellar white matter co-methylation signatures significantly associated with the degree of Purkinje cell loss, suggesting that the observed changes in DNA methylation may be playing a role in circuitry dysfunction and leading to degeneration of these cells.

An increasing number of studies reporting on epigenetic changes in neurodegenerative diseases have emerged in recent years. For example, Smith and colleagues report that elevated brain DNA methylation in the *HOXA* gene cluster on chromosome 7 is associated with AD neuropathology [45]. In line with this, our co-methylation network analysis identified a positive association between the module containing *HOXA2* and additional CpGs in that region of chromosome 7 and the MSA disease status. It is also interesting to note that a recent PSP EWAS has shown significant DNA methylation changes in *LMAN2* [52], which we also detected in MSA. Interestingly, a study aiming to identify common DNA methylation changes across neurodegenerative diseases [42] has shown shared changes in *HIP1* (AD and Down syndrome) and *MOBP* (PD and Dementia with Lewy Bodies). Furthermore, shared affected pathways include “regulation of actin cytoskeleton” and “axon guidance”. These findings, together with ours, emphasise the possibility that changes in DNA methylation profiles could be shared across neurodegenerative diseases. Moreover, our findings that deregulation of DNA methylation (such as the CpG in *LMAN2*) can occur across different brain regions with variable pathological burden, poses the question of whether these shared changes could represent general disease mechanisms that are affected in the early phases of disease pathogenesis. Altogether, these findings may be relevant for drug development, as drugs against these targets could potentially be effective against multiple neurodegenerative diseases.

We are fully aware that our EWAS study, like many others, comes with limitations. First, the potential influence of unknown genetic variants on the epigenetic patterns observed cannot be ruled out. Second, environmental factors, such as medications, smoking status, or other pathologies might as well affect DNA methylation profiles. Here, we adjusted our data for possible confounders and used stringent criteria for loci selection and statistical thresholds. Future work, and integration with methylome datasets collected by other research groups will help identify the precise contributions of all these factors to the DNA methylation profiles reported here. Another potential limitation of this study is the fact that it uses post-mortem brain samples. The observations we report here are relevant to the end stage of the disease and, therefore, causality cannot be inferred. However, having found significant DNA methylation changes in a minimally affected brain region, such as the occipital white matter in MSA, as well as changes that correlate with the severity of pathology, suggests DNA methylation at these loci is deregulated early in the disease pathogenesis. Finally, although the sample size is rather modest, our study is strengthened by its design: (a) we dissected white matter, which minimizes cell type heterogeneity, consequently reducing the noise in the data, and further accounted for possible neuronal contamination in our statistical analyses; (b) we ranked the MSA cases based on pathological subtypes to obtain homogeneous disease cohorts; (c) we performed a multi-stage study, enabling us to replicate some of the initial findings in the follow-up stage; and (d) we used two independent methodologies for data analysis (differential methylation analysis and co-methylation network analysis), and both approaches identified concordant results. In future work, it would be interesting to investigate additional brain regions affected in MSA, including the striatonigral system, pons, medulla and subcortical white matter in the motor cortical region.

Here, we have conducted the first EWAS in MSA using a multi-stage study design, which allowed us to report on consistent DNA methylation changes associated with MSA, MSA subtypes and neurodegeneration. Our data provide the first evidence for changes in DNA methylation across brain regions in MSA, including in *HIP1*, *LMAN2* and *MOBP*, all relevant to neurodegenerative diseases. Our analyses of multiple brain regions with different degrees of GCI pathology, demonstrated that some DNA methylation changes mirror the MSA-associated pathology (i.e. cerebellum-specific or at least stronger effects in the cerebellum, the most severely affected brain region analysed), such as CpGs in *SRP9* and *DGKI*. We also detected changes shared with other neurodegenerative diseases, including changes in *HIP1*, *LMAN2*, *MOBP* and in the *HOXA* gene cluster, suggesting these loci could be involved in common mechanisms implicated in neurodegeneration. Our co-methylation network analysis further complemented the results from the differential methylation analysis by implicating neurodegeneration relevant pathways, including those related to cell death signalling, Rho GTPase signalling, axon–oligodendrocyte coupling, protein quality control, neuroinflammation, and mitophagy. These findings will pave the way to more extensive research into the mechanisms leading to these changes, and may lead to improved disease classification, disease management, and novel therapeutic interventions.

## Electronic supplementary material

Below is the link to the electronic supplementary material.

Supplementary file1 (XLSX 14 kb)

Supplementary file2 (XLSX 69 kb)

Supplementary file3 (XLSX 34 kb)

Supplementary file4 (XLSX 11 kb)

Supplementary file5 (XLSX 34 kb)

Supplementary file6 (XLSX 904 kb)

Supplementary file7 (PDF 661 kb)
