# Supplementary material for: White matter DNA methylation profiling reveals deregulation of HIP1, LMAN2, MOBP, and other loci in multiple system atrophy
Source: Acta Neuropathol. 2019 Sep 18;139(1):135–56. doi: 10.1007/s00401-019-02074-0 (PMC6942018; doi:10.1007/s00401-019-02074-0)
Supplement: Supplementary file 7 — Supplementary file7 (PDF 661 kb) [file 401_2019_2074_MOESM7_ESM.pdf]

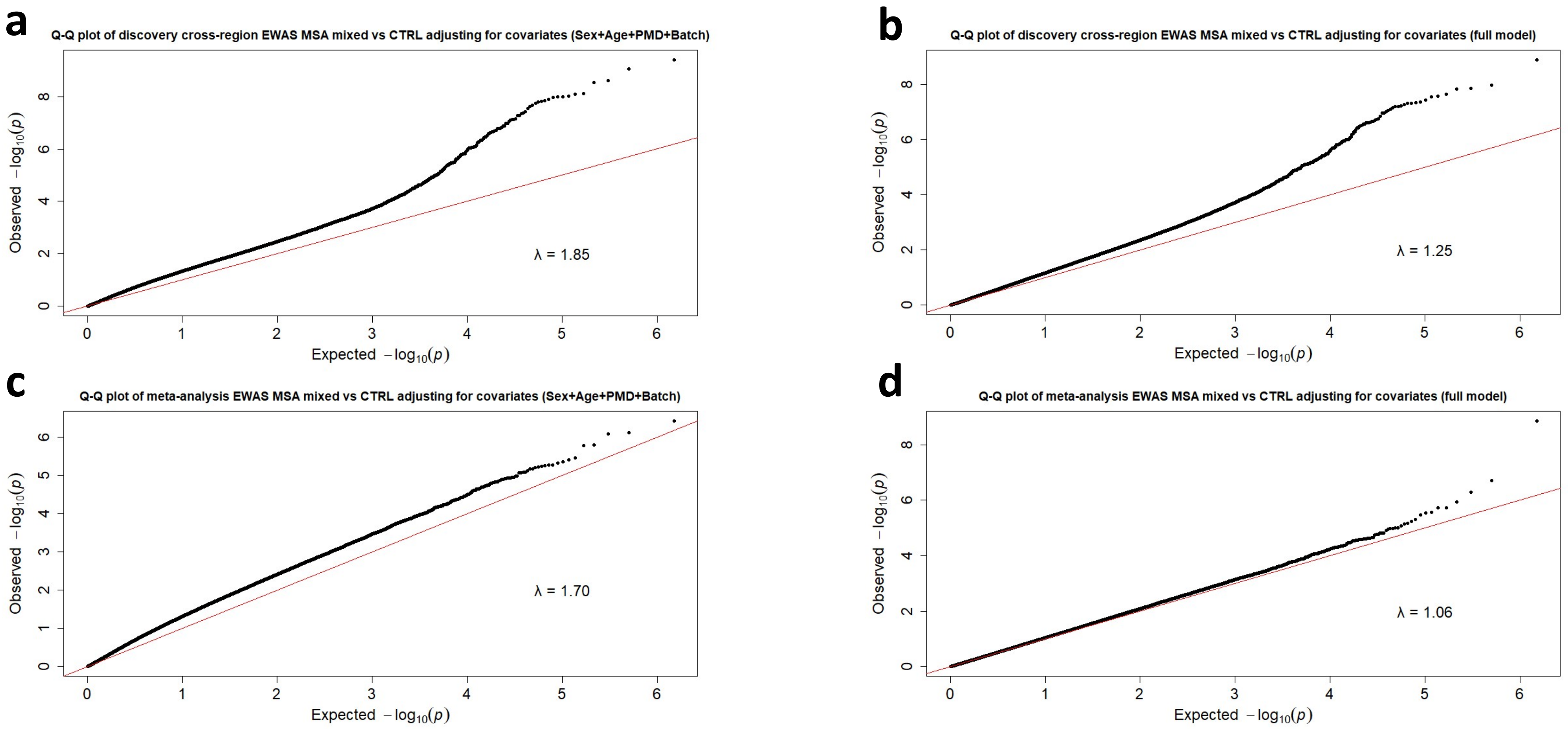

Supplementary figure 1. Quantile-quantile plots (Q-Q plots) showing the epigenome-wide p-values deviation from the expected null distribution, and the genomic inflation factor lambda (λ). **a-b)** show the results for the discovery cohort using a partial model (adjusting for sex, age, post-mortem delay (PMD), and batch effect) and the improvement when using the full model (adjusting for sex, age, PMD, batch, neuronal proportions and surrogate variables), respectively. **c-d)** show the results for the meta-analysis (all MSA mixed vs all controls) using the same partial model and the improvement when using the same full model, respectively. Note: a similar decrease (~0.6) in λ was observed in both discovery and meta-analysis when using the full model.
